# Clinical and therapeutic outcomes of COVID-19 intensive care units (ICU) patients: a retrospective study in Ghana

**DOI:** 10.11604/pamj.2021.38.107.27131

**Published:** 2021-02-02

**Authors:** Jane Afriyie-Mensah, Elvis Twumasi Aboagye, Vincent Jessey Ganu, Samuel Bondzi, Dennis Tetteh, Ernest Kwarteng, Joseph Akamah, Alfred Doku, Patrick Adjei

**Affiliations:** 1 Department of Medicine and Therapeutics, College of Health Sciences, University of Ghana Medical School, University of Ghana, Accra, Ghana,; 2 Department of Medicine, Korle-Bu Teaching Hospital, Accra, Ghana,; 3 West African Centre for Cell Biology of Infectious Pathogens, Department of Biochemistry, Cell and Molecular Biology, College of Basic and Applied Sciences, University of Ghana, Legon, Accra, Ghana

**Keywords:** COVID-19, Ghana, intensive care unit, SARS-CoV-2

## Abstract

The COVID-19 pandemic had caused significant morbidity and mortality, with over a million deaths recorded to date. Mortality recorded among severe-critically ill patients admitted to intensive care units (ICU) has been significantly high, especially in most COVID-19 epicenters. Reports on the unique clinical characteristics and outcomes from the ICU admissions are on-going with isolated studies in Africa. This study was a retrospective single-centre study involving all polymerase chain reaction (PCR) confirmed severe acute respiratory syndrome coronavirus 2 (SARS-CoV-2) patients admitted to the medical intensive care unit (MICU) of the department of medicine and therapeutics, Korle-Bu Teaching Hospital, over the period of 13^th^ April - 28^th^ June 2020. Twenty-two (22) patients in total fulfilled the inclusion criteria and are included in this report. Patients' socio-demographic characteristics, clinical and laboratory parameters outcomes as well as treatment modalities employed were extracted from their respective medical records and analyzed using STATA version 14. Dyspnoea, fever and cough were most common associated symptoms. The mean duration of admission at the ICU was 4.1 ± 3.0 days with five deaths (22.7%). About 91% (20/22) had at least one comorbidity with hypertension as the most prevalent. The median oxygen saturation/fraction of inspired oxygen (SpO_2_/FiO_2_) level was significantly higher in persons with only COVID-19 pneumonia compared to those with complicated respiratory failure (p<0.001). Six (27.3%) out of the 22 patients had non-invasive ventilation, with only 1/22 (4.5%) receiving mechanical ventilation. Although non-significant, the mean duration of ICU stay was relatively shorter in patients who received therapeutic doses of anticoagulation (p=0.32). Duration of treatment with methylprednisolone was significantly associated with patient outcomes (p=0.04) and serum ferritin levels had a tendency to negatively affect outcome (p=0.06). Clearly there are still no specific targeted medications for COVID-19 treatment, except for empirically symptoms-guided treatments and management of mild to critically ill patients. Early use of systemic corticosteroids for severe to critically ill patients in the ICU using S/F ratio and CRP levels may improve outcomes.

## Introduction

The novel severe acute respiratory syndrome coronavirus 2 (SARS-CoV-2), causing coronavirus disease 2019 (COVID-19), has fast evolved into a pandemic with unimaginable impacts on healthcare systems globally [1]. The onset of this pandemic since 29^th^ December 2019 has stretched the health systems of many countries particularly the already limited intensive care space which is immensely required to care for those with the severe and critical forms of the disease [2]. There are significant morbidity and mortality resulting from the pandemic, with over a million deaths recorded to date [3]. Although SARS-CoV-2 incidence and rate of transmission in some parts of Europe, Asia and America are yet to be stabilize, Africa among other nations experienced a relatively low incidence and mortality [4]. The clinical manifestations of COVID-19 resulting from SARS-CoV-2 infection, vary widely in severity, ranging from mild to moderate symptoms in about 80% [5], to severe-critical disease with rapid progression in about 20% of affected individuals, with a high case fatality in the latter group [5]. Early in the pandemic, it became clear that older age (60 years and above), male sex and the presence of underlying medical conditions such as cardiovascular disease, diabetes, chronic respiratory disease and cancer are major risk factors for developing severe to critical illness with an increased tendency of ICU admission and mortality [5,6]. However, these identified risk factors do not completely explain why some affected individuals experience none or mild/moderate symptoms while others become severely ill.

Severe COVID-19 is characterized by dyspnea (RR>30bpm), hypoxia with SpO_2_<93% on RA or >50% lung involvement on chest imaging and the presence of respiratory failure defined as partial pressure of oxygen/fraction of inspired oxygen (PaO_2_/FiO_2_) <300 or SpO_2_/FiO_2_<315, shock or multi-organ dysfunction defines critical illness [7]. Although acute lung injury (ALI) or acute respiratory distress syndrome (ARDS) appears to be the major cause of death, severe COVID-19 has also been linked with cardiovascular sequelae, such as myocardial injury, arrhythmias, cardiomyopathy and heart failure, acute kidney injury often requiring renal replacement therapy, neurological complications, encephalopathy and acute ischemic stroke [8]. Recent evidence has revealed that COVID-19 induced coagulopathy occurs presenting as micro- and macro-vascular thrombosis in both venous and arterial vasculature [9]. These complications account for the high mortality among patients admitted to the ICU with a variable case fatality of between 30-67%, particularly among mechanically ventilated patients [10-13]. Management strategies used in supporting ICU patients keeps evolving with the aim of optimizing care and reducing mortality. Reports on the clinical characteristics of severe to critically ill COVID-19 patients admitted to the ICU have largely been from outside Africa. With the current trend of a likely second wave of SARS-CoV-2 resurgence in Europe (for example France and England among many others) we reviewed the clinical characteristics and treatment outcomes of patients admitted to the MICU of the Korle-Bu Teaching Hospital in Ghana. Results of this review may impact both local and national clinical treatment guidelines to advance COVID-19 care.

## Methods

**Ethical approval:** the study was conducted according to the ethical guidelines of the declaration of Helsinki. The Scientific and Technical Committee (STC) and the Institutional Review Board (IRB) of the Korle-Bu Teaching Hospital approved this study (KBTH STC/IRB 000111/2020).

**Study design, site and participants:** this was a retrospective single-centre review of patients with laboratory confirmed diagnosis of SARS-CoV-2 infection, admitted to the medical intensive care unit (MICU) of the department of Medicine, Korle-Bu Teaching Hospital from 13^th^ April to 28^th^ June 2020. The MICU had since April 2020, been dedicated for the admission of COVID-19 patients presenting to the hospital with severe to critical disease. Laboratory confirmation of SARS-CoV-2 infection were conducted with the 2019-nCoV real time-polymerase chain reaction (RT-PCR) at the Noguchi Memorial Institute for Medical Research, University of Ghana, Legon, Accra, using nasopharyngeal swabs.

**Data collection:** a chart review of the clinical records of the patients were conducted. Data extraction form were used to obtain patients´ characteristics with parameters that includes patients´ socio-demographic variables, clinical characteristics and laboratory parameters. The socio-demographic characteristics extracted included age, sex and occupation. Data on clinical characteristics extracted included clinical history, co-morbidities present, signs and symptoms at admission including SpO_2_/FiO_2_ (S/F) ratio, duration on admission, initial laboratory parameters including inflammatory markers; D-dimer levels, C-reactive protein (CRP), erythrocyte sedimentation rate (ESR), ferritin, lactate dehydrogenase (LDH) and interleukin 6 (IL-6), medications administered, complications developed during admission and clinical outcomes (discharge from MICU or death). The data extracted were reviewed by two independent research investigators as part of data validation and quality checks.

**Data definitions**: a) the criteria for admission to the MICU was PCR positive SARS-CoV infection associated with: severe disease defined as having oxygen saturation (SpO_2_) less than or equal to 93% on room air with increasing oxygen demands, respiratory rate (RR) >30 bpm, >50% lung involvement on chest imaging and S/F ratio <315; critical disease characterized by organ/multi-organ dysfunction (lung plus any other) and evidence of sepsis/septic shock (using Q-sofa criteria). Raised serum inflammatory markers such as CRP, ESR, IL-6 and ferritin levels was used as a determinant of a systematic inflammatory response; b) cardiac Injury was defined by elevated troponin I levels (>0.16ng/ml); c) acute kidney Injury (AKI) was defined as abnormal urea and creatinine in a non-chronic kidney disease (CKD) patient; d) hepatic injury was defined as having deranged liver enzymes. For the pruposes of the study and in the absence of arterial blood gases (ABGs) for objective definition of ARDS, all patients with S/F ratio <311 were defined as having acute respiratory distress syndrome (ARDS) as described earlier by Bashar F *et al*. 2018 [14].

**Standard MICU treatment protocol:** MICU patients with abnormal S/F ratio (<400) ± elevated inflammatory markers (particularly CRP >70mg/L) were started on high dose IV methylprednisolone (100mg) stat and infusion of 400mg over 24 hours for day 1, subsequently given 250mg infusion daily for 5 days and/or till S/F >400: all patients with myocardial involvement also received IV methylprednisolone; all patients received Tab doxycycline 200mg stat and 100mg twice daily for 10 day, vitamin C 1000mg daily and zinc tablets 60mg daily; intravenous (IV) antibiotics were administered to severely ill patients with fever and neutrophilia; all patients received prophylactic SC clexane of 40mg daily except for those with D-dimer levels ≥5ug/ml who received therapeutic doses at 1mg/kg; in the absence of highflow oxygen, supplementary oxygen was titrated to obtain SpO_2_target of at least 94% using nasal prong, simple facemasks or non-rebreather masks. Patients who couldn´t achieve target SpO_2_ on 15L non-rebreather were put on noninvasive ventilation (CPAP) and patients with deteriorating saturations despite non-invasive ventilation were candidates for mechanical intubation.

**Statistical analysis:** the data was entered into an excel sheet and exported into STATA version 14 for all analysis. Categorical data were analyzed and presented as frequencies and percentages and compared for any significant difference using chi square or Fischer´s exact test accordingly. The Chi-square test was used to compare clinical symptoms to clinical outcomes and also to compare the effect of having a comorbidities on the clinical outcome. Continuous variables were analyzed and presented as median and interquartile ranges and means with standard deviations where appropriate. P-values less than 0.05 were considered statistically significant.

## Results

**Demographic and clinical characteristics:** a total of 22 patients were admitted to the MICU over the period with 12 (54.5%) of the patients directly admitted from other referral centers and the rest from within the hospitals´ COVID-19 treatment centre. The median age of the MICU patients was 62 years (age range of 17-81 years) with 63.6% (14/22) being at least 60 years. About 55% of patients were males. The most prevalent symptoms were dyspnoea, fever and cough (Table 1). The median SpO_2_/FiO_2_ ratio of admitted patients were 373.8 with an interquartile range of 204.2 - 433.3. Ten patients (45.5%) had S/F ratio <315 and 5 (22.7%) of patients died (Table 1). At time of analysis, one patient was still on admission with 16 (72.7%) successfully discharged from ICU.

**Table 1:** demographic and clinical characteristics of patients with COVID-19 infection admitted to the medical intensive care unit at the Korle-Bu Teaching Hospital, April - June 2020

	Frequency	Percent
**Sex**		
Female	10	45.4
Male	12	54.6
**Age (in years)**		
**Median (lower quartile, upper quartile)**	**62 (49,68)**	
**Age categories (in years)**		
<20	1	4.55
20-39	4	18.2
40-49	1	4.55
50-59	2	9.09
≥60	14	63.64
**Presenting symptoms**		
Dyspnea		
No	2	9.1
Yes	20	90.9
**Fever**		
No	4	18.2
Yes	18	81.8
**Coryza**		
No	1	4.6
Yes	21	95.4
**Myalgia**		
No	15	68.2
Yes	7	31.8
**Anorexia**		
No	16	72.7
Yes	6	27.3
**Confusion**		
No	18	81.8
Yes	4	18.2
**Altered sensorium**		
No	20	90.9
Yes	2	9.1
**Admitting SpO_2_/FiO_2_ ratio**		
Median SpO_2_/FiO_2_ ratio (LQ, UQ)	373.8 (204.2,433.3)	
**Outcome**		
Died	5	22.7
Discharged	17	77.3

**Comorbidities and duration of ICU stay:** twenty (90.9%) of the MICU patients had at least one comorbidity with hypertension being the most prevalent followed by diabetes 13 (59.1%) (Table 2). Two patients (9.1%) were newly diagnosed with HIV and only one patient was on chemotherapy for multiple myeloma. The mean duration of ICU stay was 4.1 ± 3.0 days with a range of 1 to 7 days.

**Table 2:** comorbidities among patients with COVID-19 infection admitted to the medical intensive care unit of the Korle-Bu Teaching Hospital, April - June 2020

	Frequency	Percent
**Presence of comorbidity**		
No	2	9.1
Yes	20	90.9
**Number of comorbidities**		
0	2	9.1
1	9	40.9
2	6	22.7
>2	5	27.3
**Comorbidity type**		
**Hypertension**		
No	6	27.3
Yes	16	72.7
**Diabetes mellitus**		
No	9	40.9
Yes	13	59.1
**HIV**		
No	20	90.9
Yes	2	9.1
Others*		
No	18	
Yes	6	

* Others: multiple myeloma, hemorrhoids, deep vein thrombosis, sickle cell disease, diverticular disease, stroke

**Figure 1:**
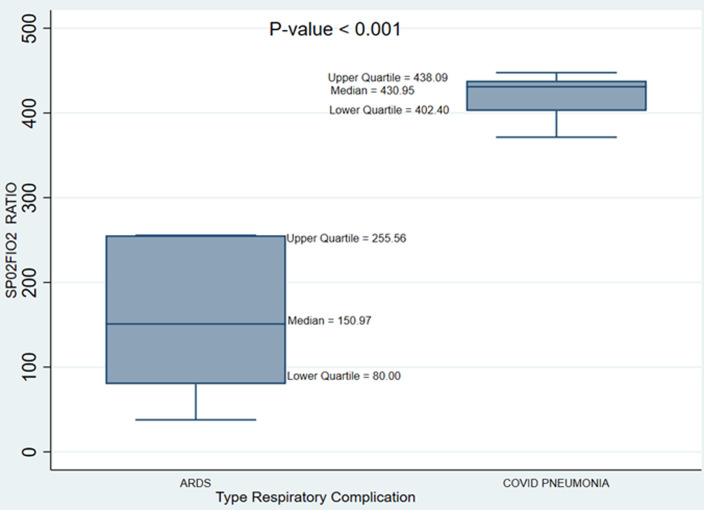
box plot showing the distribution of SpO_2_/FiO_2_ ratio by type of respiratory complication

**Complications developed:** approximately 91% (20/22) of the patients had COVID-19 related respiratory pneumonia with 54.5% having >50% lung involvement on chest computed tomography (CT)-scans. Half (10/20) of those with COVID-19 pneumonia had ARDS using S/F ratio threshold of <311 (Table 3). The S/F ratio was significantly lower in patients with uncomplicated COVID-19 pneumonia when compared to those with ARDS (Figure 1). Nine (40.9%) had neurological complications in the form of confusion, hallucination, altered sensorium and anosmia. Cardiovascular complications such as arrythmias and strokes occurred in 2 (9.1%) and 2 (9.1%) respectively and 1 patient (4.5%) had sepsis. Acute kidney Injury (AKI) occurred in 8 patients (36.4%) and acute lower gastrointestinal bleeding occurred in 2 cases (9.1%).

**Table 3:** complications among patients with COVID-19 infection admitted to the medical intensive care unit of the Korle-Bu Teaching Hospital, April - June 2020

Complication	Frequency	Percent
**Respiratory complication**		
**COVID pneumonia**		
No	2	9.1
Yes	20	90.9
**Acute respiratory distress syndrome**		
No	12	50.0
Yes	10	50.0
**Bronchospasm**		
No	21	95.5
Yes	1	4.5
**Kidney complication**		
**Acute kidney injury**		
No	14	63.6
Yes	8	36.4
**Cardiovascular complication**		
**Arrhythmias**		
No	20	90.9
Yes	2	9.1
**Stroke**		
No	20	90.9
Yes	2	9.1
**Neurological complication**		
Confusion		
No	18	81.8
Yes	4	18.2
**Altered sensorium**		
No	20	90.9
Yes	2	9.1
**Hallucination**		
No	21	95.5
Yes	1	4.5
**Gastrointestinal (GI) complication**		
**Acute lower GI bleed**		
No	20	90.9
Yes	2	9.1

**Laboratory characteristics:** the median values of haemoglobin (Hb), platelets, white blood cell (WBC) including differential cell counts were normal (Table 4). The median values of inflammatory markers such as serum ferritin, CRP, LDH and ESR were all elevated above the normal range (Table 4). C-reactive protein levels >70 was noted in 13/19 (68.4%). D-dimer levels were elevated in 19/22 patients (86.4%) with a median value of 5.0 and that of troponin T was 0.2 (Table 4). Evidence of transaminitis and cholestasis occurred in about 6/19 (32.6%) and abnormal blood urea and creatinine in 8/22 (36.4%) of the patients (Table 4).

**Table 4:** laboratory characteristics of patients with COVID-19 infection admitted to the medical intensive care unit at the Korle-Bu Teaching Hospital, April - June 2020

Characteristic	Median (LQ, UQ)
Haemoglobin	12.7 (10.1, 13.6)
Platelets	274 (206, 332)
White cell count	8.1 (5.7, 9.7)
Lymphocytes	1.8 (1.0, 2.4)
Neutrophils	5.4 (3.4, 8.6)
Ferritin	717.4 (259, 2913.2)
Lactate dehydrogenase (LDH)	465 (317, 736)
Creatinine kinase (CK)	121.5 (66, 251)
Troponin	0.2 (0.1, 0.3)
D-dimer	5.0 (0.9, 7.1)
C-reactive protein (CRP)	96 (37.7, 137)
Erythrocyte sedimentation rate (ESR)	65 (35, 90)
**Liver function tests**	**Frequency (%)**
Normal	14 (73.7)
Cholestasis	2 (10.5)
Transaminitis and cholestasis	3 (15.8)
**Renal function tests**	
Normal	14 (63.6)
Acute kidney injury	8 (36.4)

**Treatment and outcomes:** six (27.3%) had non-invasive ventilation (CPAP) and only 1/22 (4.5%) was mechanically ventilated (Table 5). All patients received anticoagulation with 8/22 (36.4%) having received therapeutic doses and the rest prophylactic doses. Approximately 73% (16/22) were given IV methylprednisolone and the mean duration of therapy was 4.9 ± 2.3 days (Table 5). For the methylprednisolone group, the mean duration of therapy was significantly shorter among non-surviving patients compared to surviving patients (p=0.04; Table 6). There was a tendency to have poor outcome with lower S/F ratio, higher serum ferritin and a higher FiO_2_ requirement although not statistically significant (p=0.07; 0.06; 0.06) respectively (Table 6). All-cause mortality rate among the ICU patients was 22.7% (5/22) (Table 1). Two (40%) of the deaths were due to acute respiratory failure whiles sepsis, acute strokes, acute lower GI bleed with hemodynamic compromise and fatal arrhythmia (ventricular fibrillation) accounted for the other three deaths. There was no significant association between the number of complications one had and the clinical outcome (χ^2^=3.82 p=0.148).

**Table 5:** treatment administered to patients with COVID-19 infection admitted to the medical intensive care unit of the Korle-Bu Teaching Hospital, April - June 2020

Treatment	Frequency	Percent
**Ventilation**		
Non-invasive		
No	16	72.7
Yes	6	27.3
Invasive		
No	21	95.5
Yes	1	4.5
Anti-coagulation		
Prophylactic	14	63.6
Therapeutic	8	36.4
**Methyl-prednisolone**		
No	6	27.3
Yes	16	72.7
Methyl-prednisolone duration (days)	4.9 ± 2.3	

**Table 6:** association between clinical outcomes and demographic, clinical and laboratory characteristics of patients with COVID-19 infection admitted to the medical intensive care unit at the Korle-Bu Teaching Hospital, April - June 2020

	Clinical outcome		
	Discharged	Died	
Characteristic	Median (LQ, UQ)	Median (LQ, UQ)	P-value
Age	62 (58, 66)	63 (49, 68)	0.97
**Clinical parameters**			
Duration in ICU	4.5 (1, 7)	2 (2, 3)	0.70
FiO_2_	0.2 (0.2, 0.4)	0.5 (0.4, 0.9)	0.06
SpO_2_/FiO_2_ Ratio	400 (255.6, 438.1)	204.2 (56.7, 255.6)	0.07
Methyl-prednisolone duration	5 (5, 7)	2 (2, 4)	0.04*
**Laboratory parameters**			
Haemoglobin	12.5 (10.2, 13.5)	13.4 (8.1, 13.6)	0.94
Platelets	274 (206, 332)	263 (228, 306)	0.91
White cell count	8.4 (4.5, 9.7)	7.3 (6.7, 8.4)	1
Lymphocytes	1.8 (1.2, 2.4)	1.1 (0.8, 2.5)	0.56
Neutrophils	5.3 (3.4, 8.6)	5.5 (5.0, 5.9)	0.61
Ferritin	496 (252.7, 1115)	2920.3 (717.4, 3000)	0.06
Lactate dehydrogenase (LDH)	427 (293, 736)	661 (464, 2017)	0.21
Creatinine kinase (CK)	123 (66, 251)	120 (120, 120)	0.86
Troponin	0.2 (0.05, 0.5)	0.2 (0.1, 0.24)	0.92
D-dimer	4.3 (0.8, 7.1)	5.8 (2.4, 6.8)	0.67
C-reactive protein (CRP)	92.6 (24, 137)	96 (96, 96)	0.71
Erythrocyte sedimentation rate (ESR)	72.5 (35, 90)	45 (4, 95)	0.71

LQ: lower quartile; UQ: upper quartile

## Discussion

This retrospective review of clinical and therapeutic an outcome of severe COVID-19 cases admitted to the ICU is timely and in line with health care system measures and preparations for the emerging second wave of the pandemic. A review suggested that the ICU mortality rate for COVID-19 patients witnessed a drop to about one-third in mid-2020 compared to the start of the pandemic, partly due to the improved hospital care and symptomatic management of the disease [15]. However, some limitation exists in the understanding of the full clinical course of the disease among the severe to critically ill and more clarity will help reduce mortality significantly especially among ICU patients. This study to the best of our knowledge is among the first observational study that has reviewed clinical outcomes of COVID-19 patients admitted to ICU in sub-Saharan Africa.

**Demographic characteristics and associated comorbidities:** the current report replicates the finding of advanced age as a significant risk factor for developing severe to critical COVID-19 and for ICU admission [5,6,11]. The MICU patients reviewed had a median age of 62 years, with more than 64% being above 60 years. In a US study by Gold *et al*. ICU admission significantly increased with age as 53.8% of all hospitalized patients above 65 years were admitted to the ICU [16]. The older age risk has been linked to the increased chance of the aged having more comorbidities compared to the younger population, hence the observed poor disease outcome. It is also thought that the medications used to manage the existing comorbidities may compromise the immune system culminating in the severe disease pattern. Other responsible factors considered include slower response to viral alerts by aging of immune cells (immune senescence) allowing for high orders of viral replications. In addition, as one ages, the thymus responsible for pumping out T-cells shrinks in size, decreasing the number of T-cells release, affecting many aspects of the general immune response action [17].

Although the study was limited by the small sample size to make any conclusions on male to female ratios, the slight male predominance (55%) observed in the current report confirms earlier suggestions of higher infection rate in men compared to women. It was thought to be too early when the gender gap emerged initially in infection rates globally. However, subsequent studies have pointed to possible factors like higher expression of angiotensin-converting enzyme-2 receptors for COVID-19 virus in males than females, as well as the sex based immunological differences [18]. A large aspect of the gender difference has been associated with lifestyle, like higher levels of smoking and drinking in men more than women. There are also the opinion that generally, women showed responsible attitude toward coronavirus during the epic period than men´s reluctance in taking the preventive measures [18].

Presence of comorbidities has been associated with disease severity, ICU admission and poor outcomes. Similarly, about 90% of all MICU patients had associated comorbidities, mainly hypertension and diabetes which have been shown to be the most common comorbidities with poor prognosis [5,6,12]. What remains to be clarified is whether the association is statistically significant or merely a confounder, since the two conditions are common medical conditions in the elderly. Other notable but less prevalent comorbidities among the MICU patients included multiple myeloma on chemotherapy, sickle cell disease and HIV which are linked with immune status suppression.

**Complications developed:** COVID-19 infection has been shown to be associated with a heightened systemic inflammatory response in a sub-group of patients who develop severe disease and is characterized by the increased release of chemokines and cytokines such as interleukins and interferons into the blood stream called the “cytokine storm” [19]. The uncontrolled release of these inflammatory mediators fuels an abnormal systemic inflammatory response similar to what occurs in other viral infections (coronaviruses and influenza). This phenomenon is life threatening as it plays a key role in the development of complications such as ARDS and multi-organ failure. Majority (91%) of MICU cases had COVID pneumonia (confirmed on high-resolution computed tomography (HRCT) with >50% lung involvement in 60% of the patients), which emphasizes that COVID-19 primarily affects the respiratory system. This explains the lower mean S/F ratio observed among the MICU patients and the predominance of dyspnea and cough as a presenting symptom in >90% of them. The Berlin criteria defines ARDS using the physiological P/F ratio of ≤300 which significantly correlates with an S/F ratio value of 311 from a large cohort study by Bashar *et al*. [14]. Using this cut off S/F ratio, 50% (10/20) of the MICU patients with COVID pneumonia had ARDS whiles the rest had uncomplicated COVID pneumonia. This proportion was lower compared to a similar study by Arentz *et al*. in which 71% of their ICU patients developed ARDS but Wu *et al*. reported a lower prevalence of ARDS being 41.8% [11-20]. It has been shown that COVID-19 related diffuse alveolar damage (DAD) presenting as acute lung injury (ALI)/ARDS appear to be the major cause of ICU admissions and mortality [11,12].

Organ dysfunction aside the lungs have been associated with severe acute respiratory viral diseases, similarly observed in COVID-19 patients with variable reported prevalence [8]. A study by Cui X *et al*. reported an AKI prevalence of 18.1% in COVID-19 patients with an increased risk in those with severe disease [21]. This was lower compared to the prevalence of 29% reported by Yang *et al*. among ICU patients with severe to critical disease [12]. The prevalence of AKI among the MICU patients was higher (36.4%) but none required dialysis. Approximately 30% of the ICU patients had evidence of liver injury with elevated transaminases and intra-hepatic cholestasis either alone or in combination. A study by Huang *et al*.reported a higher prevalence (62%) of liver dysfunction among ICU patients with severe COVID-19 disease compared to 25% in non-ICU patients [22].

Neurological complications in the form of confusion, anosmia, hallucination and altered sensorium have also been noted with varied prevalence in patients with COVID-19 [23,24]. Although Helms *et al*. reported a higher prevalence of 84% among severely ill patients, our review showed a prevalence of 40.9% having anosmia, hallucination, confusion and altered sensorium [24]. Cardiac complications in the form of arrhythmia occurred in 2 (9.1%) of the MICU patients which was lower compared to arrhythmia prevalence of 16.7% in a cohort of Chinese patients with COVID-19 [25]. The pathophysiology of cardiac complications in COVID-19 are numerous including the increased presence of cytokines and chemokines in the blood and its effect on the myocardium, hypoxia related myocardial injury and hypoxic myocardial injury [26]. Two (9.1%) of the MICU patients presented with acute ischeamic strokes. In contrast, a study by Merkler *et al*. reported that 1.6% of patients with COVID-19 visiting the emergency department (ER) department had ischeamic strokes [27]. Both patients had a medical history of hypertension and diabetes.

With increased knowledge of coagulopathies in COVID-19 patients and the associated risks of mortality, most hospital protocols sought to therapeutically anticoagulate COVID-19 patients with severe illness and high d-dimer level although no clear studies have been done to back these measures [9]. Although routine screening for deep vein thrombosis (DVTs) with Doppler scan was not done, none of the MICU patients presented clinically with suspected DVTs but presence of pulmonary embolism could not be ruled out in the absence of CT pulmonary angiograms. Acute severe lower GI bleed however occurred in 2 patients (9.1%), one of whom had a history of diverticular disease. Both patients had therapeutic anticoagulation doses which could have contributed to the acute lower GI bleed with significant hemodynamic compromise.

**Laboratory parameters and outcomes:** the MICU patients generally had normal median values of Hb, platelets, WBCs and differential cell counts. Lymphopenia is known to occur quite frequently among patients with COVID-19 disease particularly those with severe disease and strongly correlates with mortality [11,12]. However, only 5 (22.7%) of the MICU patients had lymphopenia with no statistically significant effect on patient outcomes. Systemic markers of the heightened immune response are numerous and include procalcitonin, CRP, serum ferritin, ESR and IL-6 [19]. With the exception of procalcitonin levels and IL-6 levels which were not readily available in our setting, the MICU patients had elevated median values of inflammatory markers consistent with their disease severity. The elevated inflammatory markers however, did not significantly affect disease outcome with the exception of serum ferritin which showed a tendency towards poor patient outcome (p=0.06). Abnormal cytokine release and direct viral attachment to angiotensin-converting enzyme 2 (ACE2) receptors on the vascular endothelium leads to excess release of coagulation factors promoting thrombotic complications in patients with COVID-19 [9]. Similarly, we found increased levels of D-dimer in a significant number of our patients 19/22 (86.4%) with no clinical evidence of DVT. Also, the median value of troponin T, a marker of cardiac injury was also elevated. However, none of these serum markers significantly impacted mortality in our patients, although the small study population could be a limitation.

**Outcomes and associated factors:** at the time of study, mortality rate at MICU was 22.7% with 16 (73%) of the patients successfully discharged from the MICU with the exception of one patient who was stable on minimal FiO_2_ awaiting discharge. Our overall ICU mortality rate was lower compared to 30.9% and 52.5% mortality reported by some studies [10,11]. Although about 45% of the MICU patients developed ARDS similar to 41.8% in a study by Wu *et al*. we reported 20% mortality among the ARDS patients compared to 52% in the later study. In a US study by Arentz *et al*. [20], a higher proportion of the COVID-19 cases (71%) developed ARDS with a mortality of 67%. In both studies all patients with ARDS required invasive ventilation known to be associated with a mortality of >50% [13]. As per local protocol to manage the limited ventilators available, 7 out the 10 MICU patients with ARDS were initially managed on non-invasive ventilation (NIV), with one escalated to invasive ventilation while the other three were on 15L oxygen via non-rebreather mask. The very minimal MV requirement among our cases could also have contributed to the lower mortality rate (20%) in those with ARDS, including the mechanically ventilated patient. Current observational studies have shown better outcomes in COVID-19 patients with hypoxemic respiratory failure who were initiated on non-invasive ventilation compared to those who had invasive ventilation and prevented escalation to invasive ventilation [28].

Although not statistically significant admitting FiO_2_ requirements and S/F ratio showed a tendency to affect mortality (p=0.06; p=0.07). The notable difference in the FiO_2_ requirements and S/F ratio between survivors and non-survivors, similarly, observed in a study by Yang *et al*. indicates that these parameters reliably depicts disease severity and predicts poor outcome [12]. Earlier on in the pandemic, the use of systemic corticosteroids was not recommended, however, some studies had shown mortality benefits of steroid use in COVID-19 associated ARDS, but the dose and duration of therapy was unclear [29,30]. As per the MICU protocol, IV methylprednisolone was added to the treatment protocol as an immune modulator using S/F ratio and CRP levels to categorize patients at risk of deterioration. It was observed in the current review that the duration of therapy with methylprednisolone was significantly shorter in the non-surviving MICU patients (p=0.04). This is likely a reflection of late patient transfer to the ICU as well as late initiation of immune-modulatory therapy such as methylprednisolone, which has been shown to mitigate the cytokine storm and its associated multi-organ dysfunction thereby reducing mortality among severe to critically COVID-19 patients [29,30].

**Figure 2:**
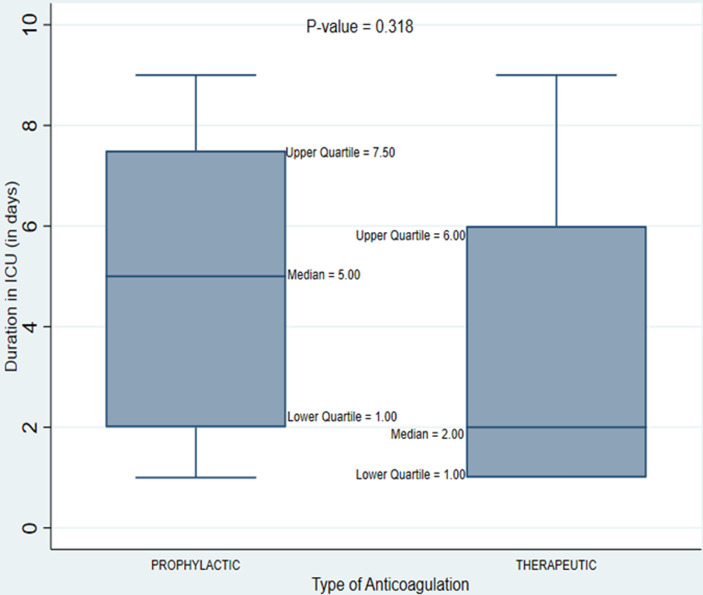
box plot showing the distribution of duration in ICU by type of anticoagulation received during ICU admission

The possible reasons for the comparatively shorter patient stay at MICU (4.1 ± 3.0) could be due to the fact that the unit had only one mechanically ventilated patient compared to other studies [10,11,20]. From the study results as shown in Figure 2, those on therapeutic dose of enoxaparin appeared to have had a shorter ICU stay compared to those on prophylactic doses, though this was not statistically significant and its use did not affect patient outcomes. Although other organ dysfunction in COVID-19 particularly the involvement of the cardiovascular system contributes significantly to mortality, our results showed no significant association between the presence of organ dysfunction and disease outcome.

## Conclusion

The early use of systemic corticosteroid therapy among MICU patients may have contributed to the relatively lower mortality rate and shorter ICU stay observed at the unit in the early months of the pandemic. We suggest the use of S/F ratio in low resource settings for early identification of COVID-19 patients at risk of severe hypoxic failure [16].

### What is known about this topic

- Patients admitted to ICU with severe/critical COVID-19 have a high mortality;
- Mortality resulting COVID-19 is highly age dependent and variations in population age, or in the age of admitted patients may have a significant influence on mortality rate.

### What this study adds

- Emphasizes the benefits of the recently recommended use of systemic corticosteroids in severe/critically ill COVID-19 patients;
- Therapeutic doses of anticoagulants administration may decrease length of ICU stay in severe/critical COVID-19 patients;
- Median SpO_2_/FiO_2_level was significantly higher in persons with only COVID-19 pneumonia compared to those with ARDS (p<0.001) and use of ratio should be encouraged in resource limited countries.
